# Determination of temperature thresholds for the parasitoid *Diachasmimorpha longicaudata* (Hymenoptera: Braconidae), using life cycle simulation modeling: Implications for effective field releases in classical biological control of fruit flies

**DOI:** 10.1371/journal.pone.0255582

**Published:** 2021-08-13

**Authors:** Shepard Ndlela, Abdelmutalab G. A. Azrag, Samira A. Mohamed

**Affiliations:** 1 Plant Health Division, International Centre of Insect Physiology and Ecology (*icipe*), Nairobi, Kenya; 2 Department of Crop Protection, Faculty of Agricultural Sciences, University of Gezira, Wad Medani, Sudan; University of Carthage, TUNISIA

## Abstract

The braconid parasitoid *Diachasmimorpha longicaudata* (Ashmead) (Hymenoptera: Braconidae) is one of the most important natural enemies in classical biological control programs against tephritid fruit flies worldwide. In light of the spread of the invasive fruit fly species, *Bactrocera dorsalis* in Africa and beyond, there is a need to implement classical biological control. The current study aimed to determine temperature thresholds for *D*. *longicaudata* reared on *B*. *dorsalis*, using life cycle simulation modeling to guide informed parasitoid releases in Africa. Simulated parameters included thermal requirements, population growth parameters at different temperature requirements, suitable areas for the establishment, and the number of generations per year under projected climatic conditions. The lower thermal threshold for the development was estimated at 10.0°C, with a thermal constant (*k*) of 333.3-degree days, while the maximum temperature threshold was estimated at 33.69°C. Fecundity was highest at 25°C, with 177.3 eggs per female. Temperature significantly affected the population growth parameters of *D*. *longicaudata*, and the maximum value of the intrinsic rate of increase (*r*_*m*_) was 0.145 at 27°C. Results indicate that *D*. *longicaudata* could successfully establish in tropical and sub-tropical regions under current and future climatic conditions. However, a slight change in the suitable areas is expected by the year 2050 due to a slight and gradual rise in temperature. Our findings provide important information for further release of this parasitoid in Africa as well as designing pest management strategies to limit the spread and reduce the impact of fruit flies sustainably.

## 1. Introduction

In the last decade, the consequences of climate change on the distribution, abundance, and phenology of insect species have been widely studied [1–3]. Various factors, chief among them; temperature, are known to affect the fitness or performance-related traits of insects [4–6]. Insect distribution and abundance are highly affected by temperature and generally, an increase in temperature within the limits tolerated by the insect result in a rapid population increase [7, 8]. It is therefore of great importance to understand how insect species respond to various climatic variables in terms of species repartition, development, survival distribution, and abundance under different climate change scenarios.

Invasive pests often arrive in new environments without their co-evolved natural enemies, hence in the absence of natural control they rapidly increase in numbers in these newly invaded areas [9, 10]. This was the case with the invasive fruit fly species *Bactrocera dorsalis* Hendel (Diptera: Tephritidae) in Africa. Following its first detection in Kenya in 2003 [11, 12], *B*. *dorsalis* rapidly invaded different ecological and climatic zones in Africa causing huge economic losses to the horticultural industry, especially for the resource-poor smallholder farmers who could not contain the devastation [13–16]. *Bactrocera dorsalis* attacks more than 100 fruit species, with mango being the most preferred host for offspring development and survival [14, 17]. In the absence of management measures, losses due to the direct feeding of the larvae on fruits and vegetables can be as high as 80%. Due to its invasiveness, the pest has been reported in Europe [18] and frequently captured in the USA [19] but without establishment.

The availability of suitable environmental conditions, unlimited host availability, and the lack of effective natural enemies (either indigenous or introduced) immensely contributed to the unchecked reproduction and spread of *B*. *dorsalis* in Africa [15, 20]. Thus, in light of the invasion by *B*. *dorsalis*, *Diachasmimorpha longicaudata* (Ashmead) (Hymenoptera: Braconidae) was imported from Hawaii and released against *B*. *dorsalis* in East Africa [21] West Africa, and Southern Africa [unpublished data] following the necessary regulatory evaluation [20]. *Diachasmimorpha longicaudata* has been extensively used in many parts of the world, in biological control programs of various Tephritid fruit flies such as *Bactrocera* sp, *Anastrepha* sp, and *Ceratitis* sp [22–26]. This parasitoid has been used in North and South America including associated islands [27, 28]. *Diachasmimorpha longicaudata* is ideal for biological control programs because of its amenability to being mass-reared on an artificial diet, its high reproductive rate, intrinsic rate of increase, and doubling time [27].

Despite the widespread use of *D*. *Longicaudata* in biological control programs, several knowledge gaps that could potentially have a huge bearing on the establishment in field conditions still exist. Studies on development in the laboratory can play a pivotal role in extrapolating to natural environments. Temperature is the most environmental factor that affects insect development and survival and reproduction [28, 29]. The ability of an insect to develop under a wide range of temperatures is an important adaptive mechanism for enhanced survival, reproduction, and growth in different climatic conditions [8]. Based on the localized success achieved when *D*. *longicaudata* was released in Kenya, Ethiopia, Benin, and Mozambique [unpublished data], efforts are currently underway to release the parasitoid in Malawi, Zambia, Zimbabwe, and other African countries. In this regard, knowledge of environmental conditions where *D*. *longicaudata* could successfully establish is essential to guide further releases in Africa and beyond. Unfortunately, the optimum temperature for the offspring development as well as the life table parameters that indicate the potential of the population growth at given environmental conditions are only partially documented. Knowledge of the thermal requirements and population growth parameters for the natural enemy is important for understanding its seasonality, population dynamics and predict the areas that are climatically suitable for its establishment. Thus, the objectives of this study were to develop a temperature-dependent development model for *D*. *longicaudata* to estimate the thermal requirements and population growth rate and project suitable areas for the parasitoid establishment to guide informed releases.

## 2. Materials and methods

### 2.1. Insect rearing

*Bactrocera dorsalis* and the parasitoid, *D*. *longicaudata* used in this study were reared at the Animal Rearing and Quarantine Unit (ARCU) of the International Centre of Insect Physiology and Ecology (*icipe*) Nairobi, Kenya. Rearing protocols for *B*. *dorsalis* were as per the procedures described by Ekesi *et al*. (2007b) [30] and Ekesi & Mohamed (2011) [31]. Larvae were raised on a carrot-based diet composed of carrot powder (24.2 g), sugar (16.2 g), brewer’s yeast (8.1 g), citric acid (0.6 g), methyl p-hydroxybenzoate (0.2 g), and water (50.7 ml). Adult flies were kept in Perspex cages of various sizes depending on experiments and maintained at 26–28°C, 60%–70% RH, and a photoperiod of 12:12 L:D. They were fed on an artificial diet consisting of a mixture of sugar and enzymatic yeast hydrolysate (USB Corporation, Cleveland, Ohio, USA) at the ratio of 3:1 by volume and were provided with water in a Petri dish (8.6 cm diameter) with a layer of pumice granules to avoid drowning. Since flies have been reared for more than 100 generations, wild flies were added to the colony at 3-month intervals to maintain genetic variability.

*Diachasmimorpha longicaudata* was reared on late second instar larvae of *B*. *dorsalis* (4-5- day-old) following procedures similar to those described by Wong & Ramadan (1992) [32] and Mohamed *et al*. (2008) [20]. Wasps were maintained at ambient conditions, 25–27°C, 60–70 RH% and photoperiod of 12:12 L:D in perspex cages (40 × 40 × 40 cm) and provided with fine drops of pure honey streaked on the topside of the cages and water on moist cotton wool balls (5–6 cm diameter). Parasitized late second instar larvae of *B*. *dorsalis* were placed in a carrot-based diet as described above. Puparia were then removed from the sand and placed in plastic basins (9.5 cm diameter, 5.5 cm deep) to allow emergence of flies and parasitoids.

### 2.2. Female oviposition and adult longevity

Female oviposition was studied in temperature-controlled incubators (Panasonic Healthcare Co., Ltd, Sakata Oizumi-Machi Ora-Gun, Gunma, Japan) set at 15, 20, 25, 30, 32.5 and 35°C, 60–70% RH and photoperiod of 12:12. L:D Soon after adult parasitoid emergence, a couple of female and male *D*. *longicaudata* were introduced into a ventilated Perspex cage (12 × 6.5 × 12 cm) and provided with fine drops of pure honey streaked on the topside of the cages and water on moist cotton wool balls (3–4 cm diameter). Each female-male parasitoid couple was provided with 25 late second instar larvae of *B*. *dorsalis* (4-5-day-old) in a carrot diet placed in oviposition units (modified petri dish, 5.5 cm in diameter by 3 mm depth) and allowed to forage and oviposit for 24 h. Thereafter, the oviposition units were removed and replaced daily with the same number of larvae until the female parasitoid died. If the male wasp died first, it was replaced immediately with an experienced one to continue mating with the female. The parasitized larvae were removed daily and dissected in saline buffer solution under a stereomicroscope (Leica EZ4D digital stereomicroscope; Leica Microsystems, Heerbrugg, Switzerland) to determine the number of parasitized host larvae as well as the number of eggs laid per female per day. The day on which the female or male parasitoid died was also recorded. Each parasitoid couple was considered to be a replicate; thus, the experiment was replicated 50–67 times at 15, 20, 25, 30, 32.5, and 35°C depending on the availability of parasitoids of similar age (minimum replication was set at 50).

To assess adult longevity newly, emerged 100 males and 100 females were separately placed into a ventilated Perspex cage (20 × 15 × 15 cm) and provided with fine drops of pure honey streaked on the topside of the cages and moist cotton wool balls as a water source. Four cages for each sex were introduced at each constant temperature of 15, 20, and 25 as replicates bringing the total number of males and females used at each temperature to 400 individuals. At 30 and 35°C, the cages were replicated six times for each sex (600 males and 600 females). Adults were observed daily and the number of dead males and females at each constant temperature were counted and recorded daily.

### 2.3. Development of immature life stages

The development duration from egg to adult (considered as the day the host larvae were parasitized to the day the adult parasitoids emerged) was determined by placing 350 late second instar larvae of *B*. *dorsalis* in oviposition units (8.5 cm by 0.3 cm, covered with organdy material) containing carrot diet and exposing them to between 300 and 350 gravid females of *D*. *longicaudata* (8-10-day-old) in perspex cages (30 cm × 30 cm × 30 cm) for an adequately short period of 4 h to allow synchronization of developmental stage duration [33]. This was carried out at ambient conditions (26–28°C, 60–70% RH, and photoperiod of 12:12 L:D). The larvae were then transferred to a fresh carrot diet in plastic basins (9.5 cm diameter, 5.5 cm deep) which were subsequently placed in a bigger plastic container (19 cm × 13 cm × 8 cm), covered with organdy material and provided with a thin layer (1 cm) of sterilized sand on which mature host larvae popping out of the diet would pupate. The above preparation was placed in incubators at 15, 20, 25, 30, and 35°C and was replicated 11 times. They were checked daily and the number of parasitoids emerging every day was recorded.

### 2.4. Model parametrization

The thermal thresholds for *D*. *longicaudata* development and survival were estimated using temperature-dependent development models. These models described the relationships between temperature and the demographic characteristics of *D*. *longicaudata*. For this purpose, the Insect Life Cycle Modelling program (ILCYM version 3.0) was used [34]. ILCYM is open-source software that has a model builder module that allows fittings of non-linear models to describe the relationships between insect demographic characteristics and temperature. The best-fitted model was selected based on statistical criteria, combined with the biological characteristics of the species (i.e. the temperature range in which immature stage survives and females produce eggs). These statistical criteria include the coefficient of determination (R^2^), which is defined as the portion of explained variation by the model, and Akaike’s Information Criterion (AIC), which is a technique based on in-sample fit to estimate the likelihood of a model to predict the future values. After building the temperature-dependent development models for *D*. *longicaudata*, all the models were compiled and used to simulate the population growth parameters that determine the population growth rate using the simulation module in ILCYM. Thereafter, we used the complied models and simulated population growth parameters to predict the protentional distribution and generations of *D*. *longicaudata* under current and future climatic conditions using population analysis and mapping module in the same program.

### 2.5. Temperature-dependent development time and adult longevity

The distribution of insect development time data was normalized using the natural logarithm (Ln) transformation. Afterward, the cumulative frequency of the development was plotted against ln-transformed development time in parallel lines, by fitting the cumulative frequency distribution. Logit function gave a better fit for total development from egg to adult and female longevity, while the complementary log-log (CLL) was the best for the male longevity. The mathematical equations of these functions are given below:

Logit distribution function: *f*(*x*) = 1/(1+exp(−(*a*_*i*_+*b* ln*x*)))

CLL distribution function: *f*(*x*) = 1−exp(− exp(*a*_*i*_+*b* ln*x*))

where *f*(*x*) is the probability of *D*. *longicaudata* to complete its development at time *x*, ln*x* is the natural logarithm of the observed development times (in days), *a*_*i*_ is the intercept corresponding to temperature *i* and *b* is the common slope of the model [34].

### 2.6. Temperature-dependent development rate and mortality

The development rate for *D*. *longicaudata* was calculated for each constant temperature as the inverse of development time (development rate = 1/developmental time). Then, the development rate was regressed against temperature and fitted to the linear model. The thermal summation approach employed here is based on the middle linear portion of the temperature-developmental rate relationship [35]. The lower thermal threshold for the development (*T*_*min*_) and the thermal constant (*k*, in degree days) were estimated from the linear model as follows:

Linear model: *r*(*T*) = *a*+*bT*

The lower thermal threshold: *T*_*min*_ = −*a*/*b*

Thermal constant: *k* = 1/*b*

where, *r*(*T*) is the development rate of *D*. *longicaudata* at temperature *T*; and *a* and *b* are the intercept and slope of the linear regression model, respectively.

However, due to the nonlinearity of the relationship especially at extreme temperatures, fifty-nine nonlinear functions embedded in ILCYM [34] were fitted to describe the relationship between the development rate and temperature. Among the fitted functions, Logan function 1 [36] was the best for the development rate of *D*. *longicaudata*. The mathematical equation for the function is given below:

Logan function 1: $r(T)=\Upsilon \left\{\exp(\rho T)-\exp(\rho \;T_{max}-\frac{\left(T_{max}-T\right)}{v})\right\}$

where *r*(*T*) is the development rate at temperature *T*; and *Υ*, *ρ* and *T*_*max*_ are the function parameters [36].

The mortality rate from egg to adult was calculated at each constant temperature and fitted to forty-five nonlinear functions embedded in ILCYM. Based on the selection criteria (R^^2^^ and AIC), Wang function 7 [37] gave the best fit to the mortality rate. The mathematical equation of the function is given below:

Wang function 7: $m(T)=1-\frac{H}{exp((1+exp(-\frac{T-T_{opt}}{Bl}))\times (1+exp(-\frac{T_{opt}-T}{Bh}))\times H)}$

Where, *m*(*T*) is the mortality rate at temperature *T*; *T*_*opt*_ is the optimum temperature, *Bl* and *H* are fitted parameters of equation [34].

### 2.7. Temperature-dependent female fecundity and adult senescence

The cumulative proportion of egg production in relation to the age of the females, also known as age-specific fecundity was described by a modified exponential function 3. The cumulative oviposition rate was plotted against normalized females’ age expressed as a ratio of age in days at each constant temperature. The following equation was used:

Exponential function 3: *f*(*x*) = 1−exp (−*aT*^*b*^)

Where, *f*(*x*) is the cumulative oviposition frequency at the normalized female age *x*; *T* is temperature; *a* and *b* are the function parameters [34].

The number of eggs per female was calculated for each constant temperature. Then, Wang function 7 was applied to describe the relationship between temperature and oviposition per female. Senescence is an age-related decrease in organism fitness and is used to measure demographic parameters such as adult survival and fertility [38]. In ILCYM, the term senescence is used instead of mortality for adults to distinguish it from juvenile mortality [34]. The adult senescence for both sexes (males and females) was calculated at each constant temperature as the inverse of adult longevity and then regressed against temperature. Afterward, the Ratkowsky function was fitted to describe the relationship between temperature and female senescence. The same function gave the best fit to the male senescence. The following mathematical expression was used:

Ratkowsky function: *s*(*T*) = *b*(*T*−*T*_*b*_)^2^

Where, *s*(*T*) is the senescence rate at temperature *T*; *b* and *T*_*b*_, are the function parameters [34].

### 2.8. Simulation of the population growth parameters

The above temperature-dependent development models for the development time, rate, mortality, fecundity, and adult senescence were compiled and used to simulate the population growth parameters for *D*. *longicaudata*. We used stochastic simulation [39] in ILCYM and a total of 200 individuals at the egg stage were used for the simulations. The simulation was conducted for six constant temperatures ranging from 15–30°C, with 3°C intervals between the temperatures, and it was replicated three times for each temperature. This sought to establish how the change in temperature by 3°C can affect *D*. *longicaudata* population growth. The following parameters were estimated: (1) the intrinsic rate of natural increase (*r*_*m*_) that determines the population’s ability to grow under specific environmental conditions (2) The gross reproductive rate (*GRR*) which is defined as the average number of daughters that female produce during her lifetime, (3) The net reproductive rate (*R*_*0*_), which is comparable to *GRR* but taking into account immature stage mortality rates, (4) the mean generation time (*Tc*), which defined as the mean time between the birth of parents and that of offspring, and (5) the doubling time (*Dt*), which define as the time required for the population to double [34, 40].

### 2.9. Prediction of suitable areas for *D*. *longicaudata* establishment and generations

To predict the suitable areas for *D*. *longicaudata* establishment and its number of generations per year under current climatic conditions (the year 2015), we used temperature data from the WorldClim version 2.1 database. We extracted monthly minimum and maximum temperatures from the database and used them for the predictions. The data are well documented by Fick and Hijmans (2017) [41] and freely available at http://www.worldclim.org/. For future climatic conditions (the year 2050), we used downscaled temperature data of the SRES-A1B [42]. The data were downscaled using WorldClim grids and freely accessible at http://www.ccafs-climate.org. Using the simulated life table parameters of *D*. *longicaudata* and temperature data obtained for the climatic databases, we calculated and mapped the establishment index (EI) and generation index (GI) for *D*. *longicaudata* in Africa under current and future climatic conditions. The EI identifies the areas that are suitable for the parasitoid to survive and become established under specific climatic conditions. Its calculation is based on the daily immature stage survival and it has a scale between 0 and 1. Scale 1 means that some individuals of each immature life stage can survive throughout the year. Otherwise, the number of days in which a single life stage would not survive are counted and divided by the number of Julian days (365). The following equation was used for the calculations:

$$EI=1-X_{k}$$

where *X*_*k*_ is the number of days within the year in which mortality in stage *k* (egg to adult) is expected to be 100% divided by the number of Julian days (365) [34].

On the other hand, GI estimates the mean number of generations that *D*. *longicaudata* might produce within a year in a given area, based on temperature. This index was calculated by averaging the sum of estimated generation time in days calculated for each Julian day. The following equation was used for the calculations:

$$GI=\frac{\sum_{i=1}^{365}365/Tc}{365}$$

where 365 is the number of days within a year (Julian days) and *Tc* is the estimated generation time in days at each Julian day *i* (*i* = 1, 2, 3,…, 365) [34].

### 2.10. Statistical analysis

The total development time and female oviposition periods as well as male and female longevity were separately subjected to Generalized Linear Model (GLM) with a Poisson distribution to test the effect of constant temperatures on these parameters. We used this model because it is recommended for count data [43] and it has been widely used in insect development time analysis. The simulated population growth parameters of *D*. *longicaudata* including the intrinsic rate of increase, gross reproduction rate, net reproduction rate, mean generation time, doubling time, and the finite rate of increase were subjected to one‐way ANOVA, after checking for normality using Shapiro–Wilk test in R software [44]. Once significant differences were detected, the means were separated using Tukey test at *α* = 0.05

## 3. Results

### 3.1. Development time and adult longevity

Temperature had a significant effect on the development time and adult longevity of *D*. *longicaudata* (Table 1). Development of *D*. *longicaudata* from egg to adult occurred at a temperature between 15 and 30°C. However, at 35°C, the parasitoid did not complete its development to the adult stage. The mean development time significantly decreased with an increase in temperature, with total development time between 17.2 and 69.1 days at 30 and 15°C, respectively (χ2 = 1513.7, df = 3773, P < 0.0001). Male longevity varied between temperatures with longest survival reported at 15°C and shortest at 35°C (χ2 = 317.98, df = 2400, P < 0.0001). Female longevity varied significantly between 2.3 and 16.8 at 35 and 15°C, respectively (χ^2^ = 2144.3, *df* = 2400, *P* < 0.0001) (Table 1). The cumulative distribution of development times from egg to adult was well described by a Logit function with intercepts ranged between -43.63 and -65.67, and a slope of 15.56 (R^2^ = 0.99, AIC = 996.4) (Table 1). By contrast, the distributions of male longevity fitted well complementary log-log (CLL) distribution function with a slope of 1.49 (R^2^ = 0.98, AIC = 3387.0) (Table 1). While the variation in female longevity was described by Logit function (R^2^ = 0.97, AIC = 3553.9) (Table 1).

**Table 1:** Development time and adult longevity (mean ± SE in days) of *Diachasmimorpha longicaudata* with the parameters (y-intercept *a*, common slope *b*) of logit and complementary log-log distributions functions fitted to the cumulative development and adult longevity at five constant temperatures.

Parameter	Temperature (°C)					Statistics		
	15	20	25	30	35	Slope (*b*)	R^2^	AIC
Total development time	*69.11± 0.36a	32.31 ± 0.11b	19.75 ± 0.05c	17.24 ± 0.09d	-	-	-	-
Male longevity	23.99 ± 0.62a	17.76 ± 0.66b	16.89 ± 0.54b	14.21 ± 0.40c	3.31 ± 0.07d	-	-	-
Female longevity	16.79 ± 0.64a	16.13 ± 0.59ab	15.29 ± 0.49b	14.07 ± 0.39b	2.49 ± .04c	-	-	-
Intercepts (*a*_*i*_)								
Total development time	-65.67 ± 0.44	-53.71 ± 0.36	-45.90 ± 0.31	-43.63 ± 0.29	-	15.56 ± 0.10	0.99	996.39
Male longevity	-4.78 ± 0.03	-4.43 ± 0.03	-4.58 ± 0.03	-4.07 ± 0.03	-1.64 ± 0.03	1.49 ± 0.01	0.98	3387.01
Female longevity	-6.08 ± 0.05	-6.04 ± 0.05	-5.91 ± 0.05	-5.78 ± 0.05	-1.16 ± 0.05	2.41 ± 0.02	0.97	3553.88

*Means in each row followed by the same letter are not significantly different (Tukey’s HSD, *P* = 0.05).

### 3.2. Temperature-dependent development rate and mortality

**Fig 1:**
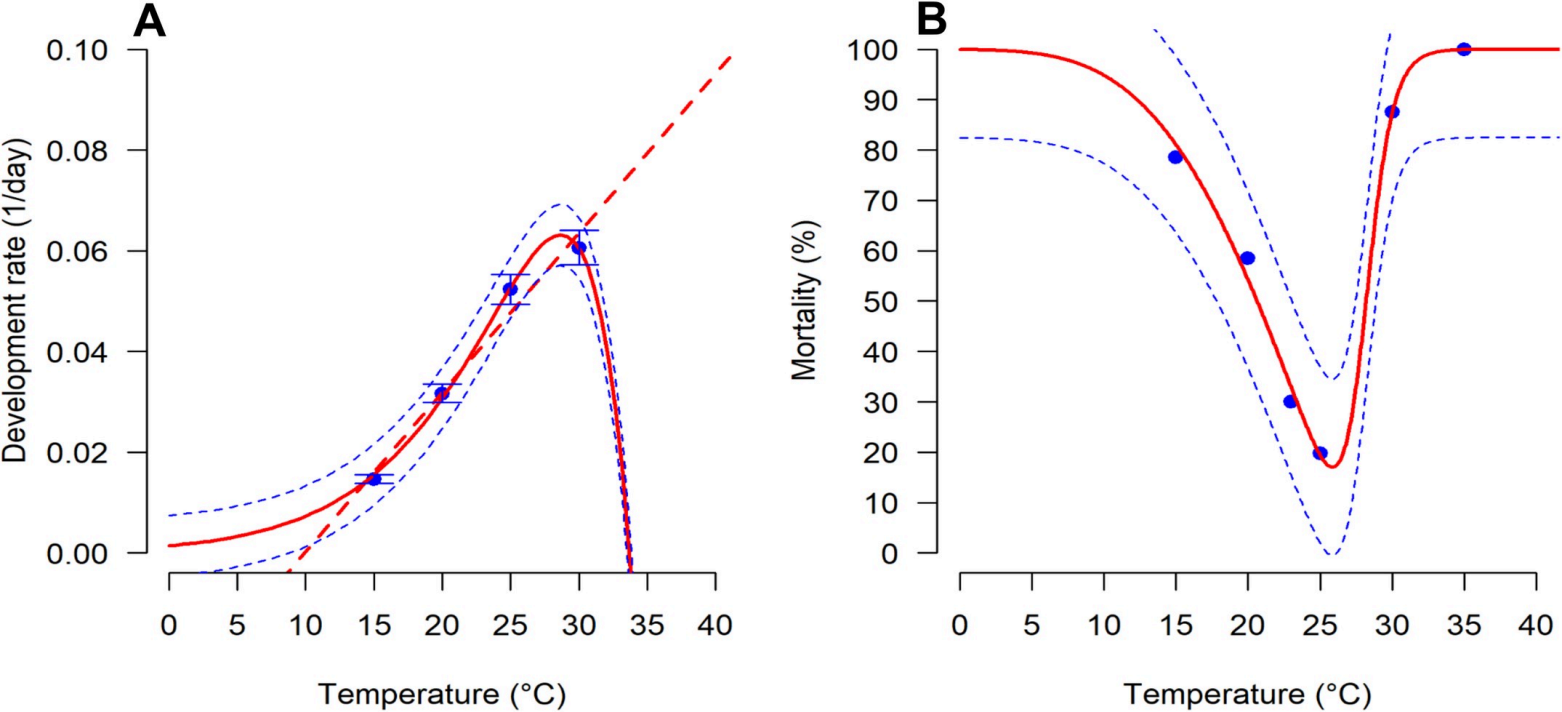
Model fitting to the relationship between (A) development rate from egg to adult and (B) mortality rate from egg to adult for *Diachasmimorpha longicaudata* reared at different constant temperatures. The blue points are the observed values of the experimental data; the dashed straight lines for linear models and solid lines for the Logan 1 and Wang 7 functions. Dashed lines in blue above and below represent the upper and lower 95% confidence interval. The experimental data for mortality was not well fitted by the model hence, we added a point at 23°C to improve model fitting [34].

Temperature had a significant effect on *D*. *longicaudata* total development rate from egg to adult (*P* < 0.05) (Table 2). The Linear model well predicted the relationship between the development rate and temperatures between 15 and 25°C (R^2^ = 0.99 and AIC = -29.60) (Table 2; Fig 1A). The lower thermal threshold for the development was estimated from the linear model at 10.0°C, with a thermal constant (*k*) of 333.3-degree days. In contrast, the Logan function 1 gave the best fit to the development rate at extreme temperatures (i.e., 30°C) (R^2^ = 0.98 and AIC = -23.43) and estimated the upper thermal threshold for the development at 33.7°C (Table 2; Fig 1A). Similarly, temperature had a significant effect on the mortality rate of *D*. *longicaudata* (*F* = 103.85; *df* = 2,3; *P* < 0.01) (Table 2). Wang function 7 well described the relationship between temperature and mortality rate with R^2^ = 0.99 and AIC = -17.99 (Table 2; Fig 1B). This function estimated an optimum temperature for the survival from egg to adult at 28.0°C, and 100% mortality occurs at 10 and 34°C, which represents the lower and upper thermal threshold for *D*. *longicaudata* survival (Fig 1B).

**Table 2:** Statistics of the goodness of fit and parameters of functions with their approximate standard errors fitted to describe the relationship between *Diachasmimorpha longicaudata* development rate and mortality rate from egg to adult and temperature.

Demographic parameters	Function name	Function parameters		Statistics				
				*F*	*df*	*P*	R^2^	AIC
Development rate	Linear	*a*	-0.03 ± 0.01	78.39	1, 2	0.012	0.97	-29.60
		*b*	0.003 ± 0.00					
		*T*_*min*_	10.00					
		*k* (*DD*)	333.33					
	Logan 1	*Υ*	0.003 ± 0.00	187.16	3, 8	< 0.0001	0.98	-23.43
		*T*_*max*_	33.69 ± 0.01					
		*ρ*	0.19 ± 1.72					
		*v*	4.72 ± 0.03					
Mortality rate	Wang 7	*Bl*	12.88 ± 1.9	103.85	2, 3	0.009	0.99	-17.99
		*Bh*	0.90 ± 0.46					
		*T*_*opt*_	28.01 ± 1.09					
		*H*	8.03 ± 0.45					

*F*: F-test statistic, *df*: degree of freedom, *P*: probability value, R^2^: coefficient of determination, and AIC: Akaike’s Information Criterion.

### 3.3. Oviposition, fecundity and adult senescence

Temperature significantly influenced female oviposition and fecundity in *D*. *longicaudata*. Pre-oviposition period was significantly longer at 15°C and shortened significantly as temperature increased (χ^2^ = 397.29, *df* = 201, *P* < 0.0001) (Table 3). Similarly, the oviposition period was longest at lower temperatures and decreased with increasing temperature (χ^2^ = 209.37, *df* = 201, *P* < 0.0001) (Table 3), while post-oviposition duration was longest at 32.5°C and lowest at 25 and 30°C (χ^2^ = 169.83, *df* = 201, *P* < 0.0001) (Table 3).

**Table 3:** Pre-oviposition, oviposition, and post-oviposition durations (mean ± SE in days) for *Diachasmimorpha longicaudata* at different temperatures.

Temperature (°C)	Pre-oviposition	Oviposition	Post-oviposition
15	6.32 ± 1.16a	23.81 ± 2.97a	5.09 ± 0.93ab
20	2.15 ± 0.41b	17.625 ± 1.39ab	2.75 ± 0.66bc
25	0.77 ± 0.24c	15.38 ± 0.95bc	1.83 ± 0.39c
30	0.15 ± 0.06d	13.00 ± 0.64c	1.59 ± 0.30c
32.5	0.41 ± 0.15cd	6.52 ± 0.73d	6.55 ± 0.89a
35	0.18 ± 0.07d	3.29 ± 0.36e	5.71 ± 0.68ab

*Means in each column followed by the same letter are not significantly different (Tukey’s HSD, *P* = 0.05).

**Fig 2:**
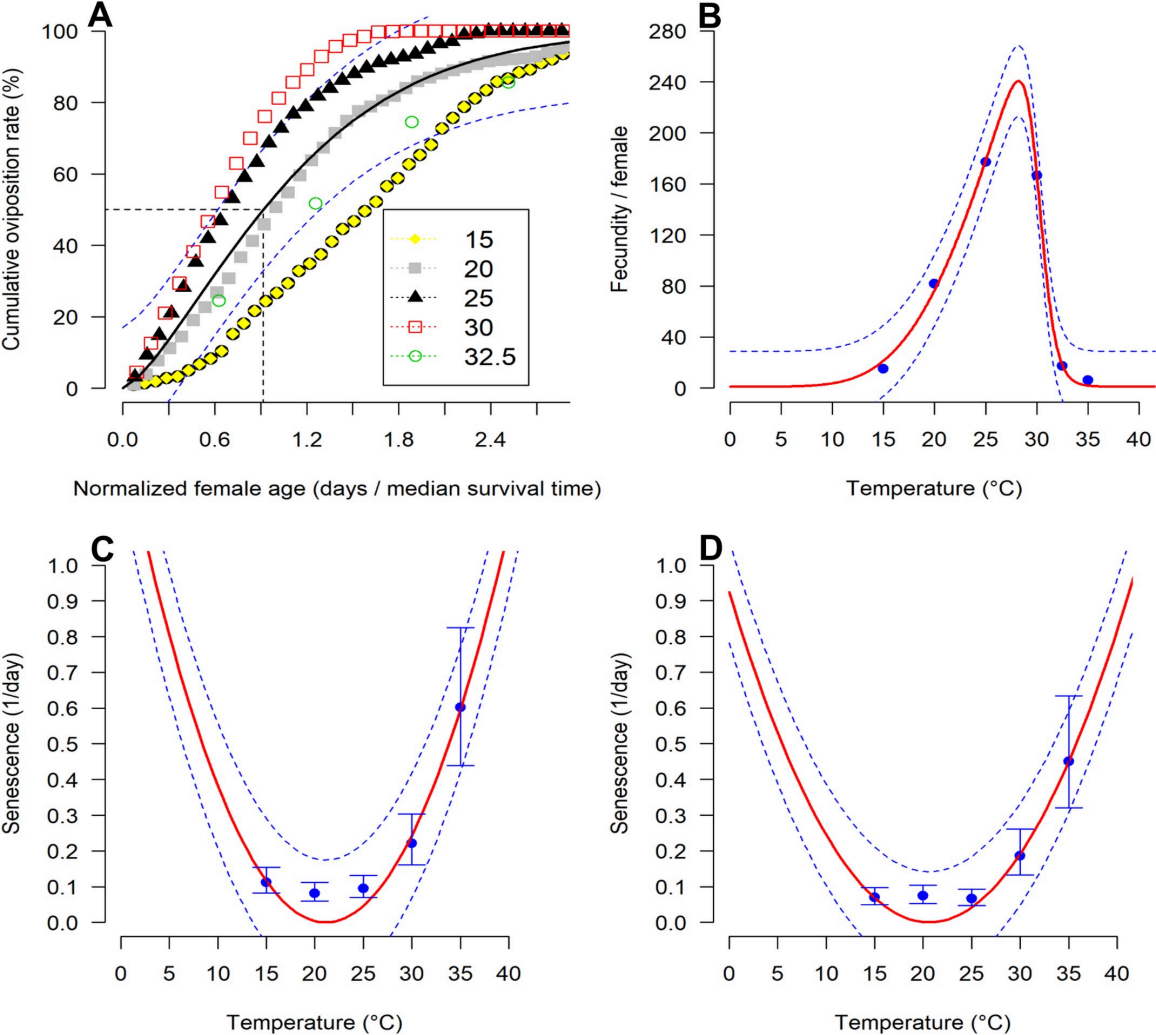
Model fitting to the relationship between fecundity in *Diachasmimorpha longicaudata* female and temperature, and adult senescence and temperature: (A) cumulative oviposition fitted to exponential modified 3 function; (B) Number of eggs per female fitted to Wang function7; (C) female senescence rates fitted to Ratkowsky function 4 model and D) male senescence rates fitted to Ratkowsky model. The observed experimental values are represented in blue points with bars representing the standard deviation. The solid red lines are the fitted models with dashed lines in blue above and below representing the upper and lower 95% confidence interval.

**Fig 3:**
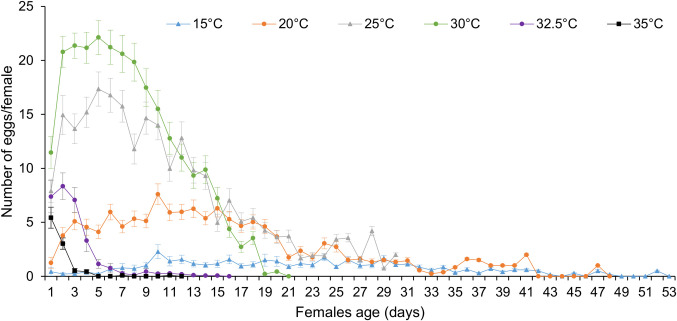
Number of eggs produced per female at different temperatures.

The exponential modified function gave the best fit to the age-specific cumulative oviposition rate (R^2^ = 0.87 and AIC = -759.82) (Table 4; Fig 2A). Fecundity was highest at 25°C, with 177.3 eggs per female, whereas the lowest fecundity was 6.3 eggs per female at 35°C (Fig 2B). However, in the first 7 days of oviposition, fecundity was highest at 30°C followed by 25°C (Fig 3).

**Table 4:** Statistics of the goodness of fit and parameters of functions with their approximate standard errors fitted to describe the relationship between *Diachasmimorpha longicaudata* cumulative oviposition, mean oviposition, and adult senescence and temperature.

Demographic parameters	Function name	Function parameters		Statistics				
				*F*	*df*	*P*	R^2^	AIC
Relative oviposition	Exponential modified 3	*a*	0.73 ± 0.03	2724.25	1, 403	< 0.0001	0.87	-759.82
		*b*	1.35± 0.06					
Mean total oviposition	Wang 7	*T*_*op*_	30.11± 0.11	243.78	3, 2	0.004	0.99	42.79
		*B*_1_	11.48± 0.62					
		*Bh*	0.78 ± 0.10					
		*H*	-2304.974±114.52					
Female senescence	Ratkowsky	*b*	0.0031 ± 0.00	60.81	1, 3	0.004	0.95	-11.39
		*T*_*b*_	21.12 ± 0.89					
Male senescence		*b*	0.0022 ± 0.00	51.49	1, 3	0.005	0.95	-13.44
		*T*_*b*_	20.61 ± 1.14					

*F*: F-test statistic, *df*: degree of freedom, *P*: probability value, R^2^: coefficient of determination, and AIC: Akaike’s Information Criterion.

The Wang function 7 gave the best fit to the relationship between temperature and female fecundity (R^2^ = 0.99 and AIC = 42.79) (Table 4; Fig 2B). The function predicted that females of *D*. *longicaudata* might be able to lay eggs under a temperature range between 10 and 36°C, with a maximum fecundity at around 28°C (Fig 2B). The adult senescence of both males and females was significantly influenced by temperature (*P* < 0.05). The effect of temperature on *D*. *longicaudata* female and male senescence was predicted by Ratkowsky function (R^2^ = 0.95 and AIC = -11.38) (Table 4; Fig 2C). The same function gave the best fit for the relationship between temperature and males senescence (R^2^ = 0.94 and AIC = -13.44) (Table 4; Fig 2D). The senescence rate for both males and females was low at 15°C and increased gradually with an increase in temperature (Fig 2C and 2D).

### 3.4. Population growth parameters

Temperature had a significant effect on the simulated population growth parameters of *D*. *longicaudata* (Table 5). The simulated intrinsic rate of natural increase (*r*_*m*_) that determines the population growth rate significantly increased as an increase in temperature between 15 and 30°C (*P* < 0.0001). The *r*_*m*_ increases significantly with an increase in temperature between 15 and 27°C (Table 5). The gross reproductive rate (*GRR*) significantly varied between 11.7 daughters per female at 15°C to 152.9 daughters per female at 27°C (Table 5). The net reproductive rate (*R*_*0*_) was maximal at 27°C, while it was the lowest at 15°C. The mean generation time (*Tc*) decreases significantly with an increase in temperature, with the longest time at 15°C, and the shortest at 30°C (*P* < 0.0001) (Table 5). The time required for the *D*. *longicaudata* population to double (*D*_*t*_) ranged between 4.8 days at 27°C and 163.9 days at 15°C. The finite rate of increase λ ranged between 1.0 and 1.2 at a temperature range between 15 and 30°C (Table 5).

**Table 5:** Simulated life table parameters (mean ± SE) of *Diachasmimorpha longicaudata* at different constant temperatures (number of eggs used for the simulation = 200). *r*_*m*_: intrinsic rate of natural increase, *GRR*: gross reproduction rate, *R*_*0*_: net reproduction rate, *Tc*: mean generation time (in days), *D*_*t*_: doubling time (in days), and *λ*: finite rate of increase.

Temperature (°C)	*r*_*m*_	*GRR*	*R*_*0*_	*Tc*	*D*_*t*_	*λ*
15	*0.003 ± 0.001f	11.722 ± 1.232e	1.259 ± 0 .053e	69.576 ± 0.557a	163.856 ± 0.000a	1.00 ± 0.001a
18	0.026 ± 0.000e	17.481 ± 1.113 de	4.235 ± 0.058d	55.358 ± 0.239b	26.592 ± 0.311b	1.03 ± 0.000b
21	0.052 ± 0.000d	29.659 ± 0.676d	13.143 ± 0.157c	49.288 ± 0.223c	13.265 ± 0.112c	1.05 ± 0.000c
24	0.092 ± 0.001b	72.355 ± 1.639c	32.58 ± 0.756b	38.047 ± 0.075d	7.572 ± 0.061e	1.09 ± 0.001e
27	0.145 ± 0.001a	152.945 ± 3.229a	42.510 ± 0.785a	25.796 ± 0.153e	4.769 ± 0.041f	1.07 ± 0.001f
30	0.067 ± 0.004c	88.016 ± 6.655b	4.085 ± 0.306d	20.903 ± 0.249f	10.402 ± 0.584d	1.16 ± 0.004d
Statistics						
*F*	968.5	289.2	1357.3	4028.0	51060.0	973.0
*df*	5, 12	5, 12	5, 12	5, 12	5, 12	5, 12
*P*	<0.0001	<0.0001	<0.0001	<0.0001	<0.0001	<0.0001

*Means in each column followed by the same letter are not significantly different (Tukey’s HSD, *P* = 0.05).

### 3.5. Suitable areas for the establishment under current and future climatic conditions

**Fig 4:**
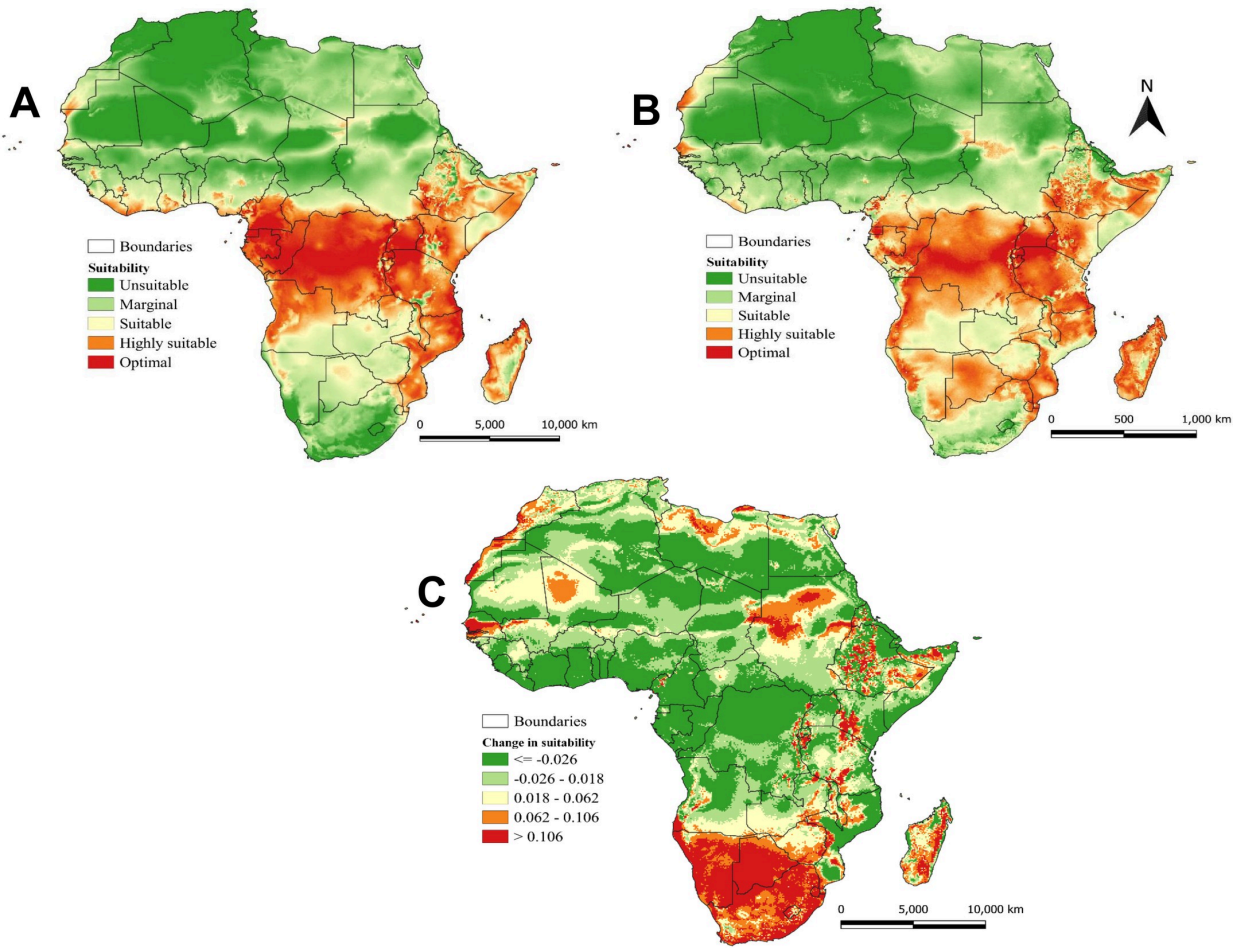
Establishment index (climate suitability) of *Diachasmimorpha longicaudata* under (A) current (the year 2015), (B) future (the year 2050), and (C) the change in establishment index between current and future climatic conditions predicted using the ILCYM model. The Establishment index is identifying the areas in which *D*. *longicaudata* may survive and become established. Indices < 0.2 indicate unsuitable areas, 0.2–0.4 for marginally suitable areas, 0.4–0.6 for suitable areas, 0.6–0.8 for the highly suitable areas, and >0.8 for the optimal areas. Republished from Fick *et al*. 2017 [41] under a CC BY license, with permission from Royal Meteorological Society original copyright ©Copyright 2020, worldclim.org.

The establishment index (EI) showing areas that are climatically suitable for *D*. *longicaudata* establishment under current and future climatic conditions is shown in Fig 4. We categorized the suitable areas for *D*. *longicaudata* establishment according to ILCYM into five levels: EI of < 0.3 indicates unsuitable areas, 0.3–0.45 for marginally suitable areas, 0.45–0.6 for suitable areas, 0.6–0.8 for the highly suitable areas, and >0.8 for the optimal areas. The areas with an EI of > 0.6 (highly suitable and optimal areas) indicate that *D*. *longicaudata* can permanently establish throughout the year. The prediction from ILCYM under current temperature conditions showed that *D*. *longicaudata* could potentially establish in most countries of sub-Saharan Africa (Fig 4A). East African countries including Kenya where *D*. *longicaudata* was released for the first time in the continent and some Southern African countries such as Mozambique, Malawi, and Madagascar showed optimal suitability (EI > 0.8) for the parasitoid establishment under current temperature conditions. Also, the model prediction showed that *D*. *longicaudata* could thrive better in Central African countries including the Democratic Republic of Congo, Republic of Congo, Cameroon, Angola, Equatorial Guinea, and Gabon.

Under probable future temperature conditions (2050), the model prediction indicates that *D*. *longicaudata* will expand its range to new areas that were previously not suitable, highly suitable, or optimal under the current climate conditions (Fig 4B). For instance, the model revealed that South African countries especially Zambia, Zimbabwe, Botswana, Namibia, Lesotho, and South Africa will be more suitable for *D*. *longicaudata* establishment, compared to the current climate scenarios (Fig 4B). In these countries, an increase in EI between 0.06–0.2 is expected, under future temperature conditions (Fig 4C). A similar increase is also expected in some parts of Sudan, Ethiopia, Kenya, and Tanzania in East Africa. However, a decrease in EI between 0.06–0.1 is also expected in some parts of these countries in the future. Gabon and Cameroon and some parts of Malawi, Nigeria, Benin, Ghana, and Ivory Coast might experience a decrease in suitability for *D*. *longicaudata* establishment (decrease in EI between 0.06–0.1) by the year 2050.

### 3.6. Generations of *D*. *longicaudata* under current and future climatic conditions

**Fig 5:**
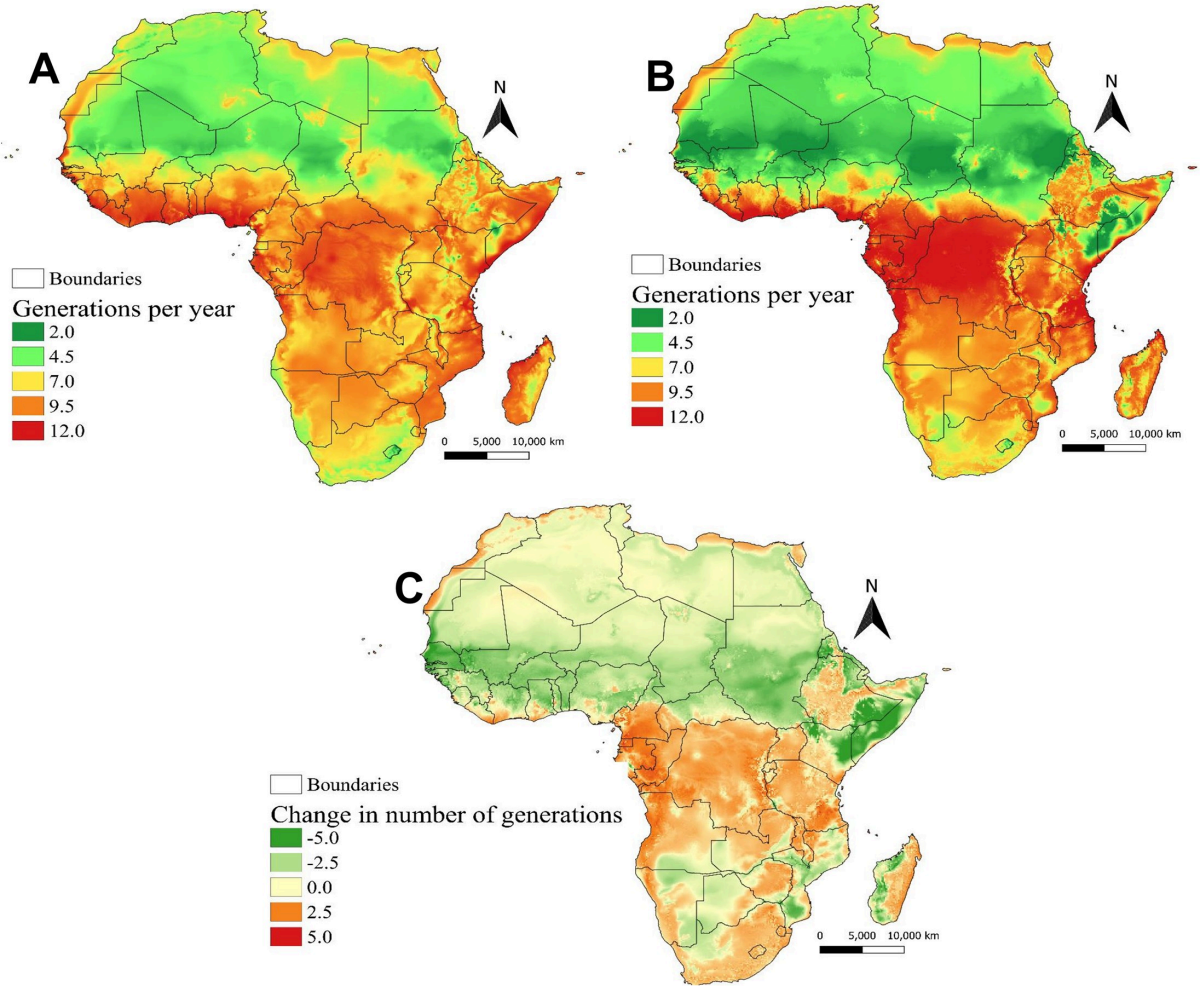
Generation index represented as the mean number of generations that *Diachasmimorpha longicaudata* might produce per year under (A) current (the year 2015), (B) future (the year 2050), and (C) the change in generation index between current and future climatic conditions predicted worldwide using ILCYM model. Republished from Fick *et al*. 2017 [41] under a CC BY license, with permission from Royal Meteorological Society original copyright ©Copyright 2020, worldclim.org.

The generation index which indicates the mean number of generations that *D*. *longicaudata* may produce within a year is given in Fig 5. Under current climatic conditions, the model estimated only 4–7 generations per year in North Africa, and between 7–12 generations in sub-Saharan Africa, especially in East and Central African countries (Fig 5A). Under future climatic conditions, more generations are expected in East, Central, and Southern African countries (Fig 5B). Due to the change in climate suitability from suitable to be marginally suitable in Sudan, South Sudan, Chad, Egypt, Libya, and some parts of Kenya and Somalia, the number of generations might be decreasing to 4 per year under future climatic conditions (Fig 5B). Change in *D*. *longicaudata* generation index is also expected between current and future climatic conditions (Fig 5C). A decrease between 2–4 generations per year is estimated by the model for Central and West African countries (Fig 5C). Similarly, some parts of Ethiopia, Kenya, Somalia, Madagascar, and Mozambique might experience a decrease in the number of generations under future climatic conditions (Fig 5C). However, in East, Southern, and some central African countries, *D*. *longicaudata* might produce an additional 1–6 generations by the year 2050 (Fig 5C).

## 4. Discussion

We report the simulations of various temperature-dependent parameters of an important parasitoid of tephritids, *D*. *longicaudata*, to guide mass rearing and field releases of the parasitoid in Africa. To adequately address and discuss the effect of climate change on determining suitable areas for field releases and parasitoid establishment, we offer validation of developmental thresholds under various temperature regimes. To the best of our knowledge, three studies have attempted to determine the effect of temperature on *D*. *longicaudata* albeit under various conditions of host species, host stage, and diet among others. Liu *et al*. 2012 [45] conducted their investigations on third instar larvae of *B*. *dorsalis* but unfortunately in our experience, exposing larvae of this stage to gravid females of *D*. *longicaudata* results in encapsulation or poor parasitoid emergence [unpublished data]. Studies of Appiah *et al*. 2013 [46] did not predict suitable areas of establishment to guide field releases. Lastly, Meirelles *et al*. 2015 [47] conducted their experiments on third instar larvae of *A*. *fraterculus* (Wiedemann) (Diptera: Tephritidae) and *C*. *capitata* (Wiedemann) (Diptera: Tephritidae). The present study showed that *D*. *longicaudata* reared on late second instars of *B*. *dorsalis* could complete its life cycle at constant temperatures ranging between 15 and 30°C, with a complete development time of 69.1 days at 15°C and 17.2 days at 30°C. These development times were longer than those obtained by Liu *et al*. 2012 [45] who reported 45.7 and 15.2 days at constant temperatures of 15 and 30°C, respectively when *D*. *longicaudata* was reared on third instar larvae of *B*. *dorsalis*. The differences between the two studies may be due to the different diets, larval instars, and methods used or the environmental conditions in which these experiments were conducted. It has been demonstrated that rearing *D*. *longicaudata* on different hosts has an effect on its developmental time [47]. However, our findings regarding developmental time were still 3 days longer at 15°C and comparative at 30°C with results of Appiah *et al*. (2013) [46]

Although insects do not develop at a constant temperature in nature, temperature-dependent development models built from the experimental data provide biological information that can be used for understanding the distribution, seasonality, and population dynamics of the insect species [48, 49]. For the natural enemies, knowledge of temperatures that favor or limit the development and population growth for the predator/parasitoid, as well as the areas where the natural enemy can establish are important in biological control programs. In this context, we described the relationship between *D*. *longicaudata* development and temperature using thermal response curves, also known as thermal reaction norms. Although the thermal response curve for an insect has a complex shape, the appropriate model that describes it should be based on the unimodal shape that predicts the minimum, the optimal, and the maximum temperature thresholds [50]. We predicted the minimum temperature threshold for *D*. *longicaudata* development at 10°C, with slight deviations to findings by Appiah *et al*. (2013) [46] who obtained 10.4°C. However, the maximum temperature threshold we obtained (33.7°C) was higher by 2.7°C, than those obtained by Appiah *et al*. (2013) [46]. These differences might be due to the different nonlinear models that were used to predict the maximum temperature threshold. We used the Logan model that considers enzyme-catalyzed biochemical reaction rate at the optimum temperature [36] compared to the Briere-2 model that was used in the study of Appiah *et al*. (2013) [46]. The Logan model has been used widely to predict the development of many tropical species such as stem borers, *Busseola fusca* F. and *Sesamia calamistis* H. [51] coffee pests *Antestiopsis thunbergii* G. and *Hypothenemus hampei* F. [6, 48] and diamondback moth, *Plutella xylostella* L. [52]. For the mortality rate, Wang 7 function estimated the temperatures at which 100% mortality occurs at 10 and 34°C, with 28°C being the optimal temperature for survival.

The adult longevity and female fecundity were significantly affected by temperature in this study. Females of *D*. *longicaudata* were found to live less than males and similar differences in adult longevity between sexes were reported by Liu *et al*. (2012) [45] and Appiah *et al*. (2013) [46]. Nevertheless, the longevity we obtained in this study at temperatures between 15–35°C (2.5–16.8 and 3.3–23.9 days for females and males, respectively) were much shorter than the 8.3–142.7 days for females and 5.7–119 days for males previously reported at the same temperature range [46]. The differences between these studies might be due to the food source used to feed *D*. *longicaudata* adults after emergence. In the present study, a highly temperature-dependent response was observed for the fecundity of *D*. *longicaudata*. A maximum fecundity of 177 eggs per female was observed at 25°C. This was 2 times higher than that reported when the host were third instar larvae of *Anastrepha fraterculus* and *Ceratitis capitata* [45]. Wang 7 function predicted that *D*. *longicaudata* could lay a maximum of ≈ 220 eggs per female at 27°C. Indeed, 27°C was predicted to be the optimal temperature for the development of the immature stage and this might explain the high fecundity at this temperature. Similarly, our result at 30°C (166 eggs per female) was 20 times higher than that reported by Liu *et al*. (2012) [45] who recorded 8 eggs per female at the same temperature. This could be an indication of *B*. *dorsalis* being a better host than both *A*. *fraterculus* and *C*. *capitata*. Furthermore, the difference between the two studies could be linked to the differences in the rearing conditions such as adult nutrition, light intensity, age of stock culture, the strain of the parasitoid population, and the density of adults in rearing cages.

The current study reported the population growth parameters of *D*. *longicaudata* which will be invaluable in elucidating its distribution, seasonality, and population dynamics. The net reproductive rate (*R*_*0*_) which indicates the population growth rate from a generation to the next one was maximal at 27°C, with 32.58 daughters per female per generation. This was higher than that reported by Liu *et al*. (2012) [45] who obtained 14.9 daughters per female per generation for *D*. *longicaudata* at the same temperature. According to life theory, if *R*_*0*_ is less than 1 in a specific environmental condition, it results in negative population growth [53]. In our study, however, *R*_*0*_ was greater than 1 at temperatures between 15–30°C, with the intrinsic rate of increase (*r*_*m*_) ranging between 0.003 and 0.15. This indicates that *D*. *longicaudata* population increase over time at a wide range of temperatures and this might be one of the factors that contributed to the success of *D*. *longicaudata* in classical biological control programs against tephritid fruit flies worldwide. Previous studies reported that the larval stage of the invasive species *B*. *dorsalis* develops at temperatures between 15–30°C [54]. Thus, *D*. *longicaudata* is expected to effectively parasitize and successfully develop on *B*. *dorsalis* larvae within the temperature range of 15–30°C. However, temperatures above 30°C and below 15°C might lower the population growth of this parasitoid, and thus affect its ability to control fruit flies in the field.

*Diachasmimorpha longicaudata* was introduced into several countries in tropical and subtropical regions to control tephritid fruit flies. Most notably *D*. *longicaudata* was released in Hawaii to control *B*. *dorsalis* [24, 55], French Polynesia [56] Pacific-South America [57–59], on *Anastrepha* sp., North America-Florida on *Anastrepha* sp. [60], and in North Africa [26] on *C*. *capitata*. However, the areas suitable for *D*. *longicaudata* establishment under different climatic conditions are still unknown for further release in Africa and beyond. In the current study, we projected the first detailed suitable areas for *D*. *longicaudata* establishment and its number of generations per year using temperature-dependent development models. The models predicted that *D*. *longicaudata* is more tolerant to a wider range of temperatures and it can be well established and survive throughout the year in sub-Saharan Africa. This indicates that *D*. *longicaudata* could well establish in the tropical regions where *B*. *dorsalis* was reported to cause high economic loss [15, 61, 62]. The projected suitable areas for *D*. *longicaudata* establishment in this study under current and future climatic conditions were similar to those projected for *B*. *dorsalis* in Africa [15, 61, 62]. Thus, *D*. *longicaudata* could well establish in areas where *B*. *dorsalis* was reported, and it will be able to follow the range expansion of *B*. *dorsalis* under future climatic conditions.

The generation index revealed that *D*. *longicaudata* is capable of completing between 9–12 generations in a year, in highly suitable and optimal areas. This finding is close to that reported by Liu *et al*. (2012) [45] though in different hosts, who obtained a mean generation time of 23–36 days (≈10–15 generations per year) at temperatures between 18–24°C. Nevertheless, change in generation index is expected due to temperature rise that will shorten the mean generation time of *D*. *longicaudata* resulting in more generations per year. On the other hand, temperature rise might also result in a decline in the number of generations per year in some areas due to unsuitable climatic conditions for *D*. *longicaudata* survival. For example, an additional 1–5 generations per year are expected in countries including Tanzania, Uganda, Democratic Republic of Congo, Congo, Gabon, Cameroon, Angola, Zambia, Zimbabwe, and South Africa. In contrast, a decline of between 1–5 generations per year is expected in North and West Africa.

## 5. Conclusions

In conclusion, the present paper validates previous findings and further provides a prediction on the establishment that may be useful when planning the release of *D*. *longicaudata* in different areas of the African continent. This information may be used in conjunction with models predicting the current and potential distribution of the host fruit flies. Simulated parameters such as mortality, longevity, rate of development, and fecundity have a huge effect on parasitoid population structure, spatial heterogeneity, dispersal, and overall establishment, contribute to the body of knowledge of efforts toward classical biological control. Our findings further reveal that *D*. *longicaudata* is tolerant to a wide range of climatic conditions. Coupled with the fact that suitable areas of the establishment will expand in the future, the parasitoid represents an excellent candidate for classical biological control of *B*. *dorsalis*.

## Supporting information

S1 Table Life table data of *Diachasmimorpha longicaudata* collected at different constant temperatures. (XLSX)
